# TIM-3 as a promising target for cancer immunotherapy in a wide range of tumors

**DOI:** 10.1007/s00262-023-03516-1

**Published:** 2023-08-11

**Authors:** Natalia Sauer, Natalia Janicka, Wojciech Szlasa, Bartłomiej Skinderowicz, Katarzyna Kołodzińska, Wioletta Dwernicka, Małgorzata Oślizło, Julita Kulbacka, Vitalij Novickij, Katarzyna Karłowicz-Bodalska

**Affiliations:** 1 Faculty of Pharmacy, Wroclaw Medical University, Wroclaw, Poland; 2 Faculty of Medicine, Wroclaw Medical University, Wroclaw, Poland; 3 Faculty of Chemistry, Wroclaw University of Science and Technology, Wroclaw, Poland; 4 Faculty of Medicine, Poznan University of Medical Sciences, Poznan, Poland; 5 State Research Institute Centre for Innovative Medicine, Department of Immunology, Vilnius, Lithuania; 6 Department of Molecular and Cellular Biology, Faculty of Pharmacy, Wroclaw Medical University, Wroclaw, Poland; 7 Faculty of Electronics, Vilnius Gediminas Technical University, Vilnius, Lithuania; 8 Department of Drug Technology, Faculty of Pharmacy, Wroclaw Medical University, Wroclaw, Poland

**Keywords:** Cancer, Immunotherapy, TIM-3, T-cells

## Abstract

T-cell immunoglobulin and mucin domain-containing protein 3 (TIM-3) expression has been a trending topic in recent years due to its differential expression in a wide range of neoplasms. TIM-3 is one of the key immune checkpoint receptors that interact with GAL-9, PtdSer, HMGB1 and CEACAM1. Initially identified on the surface of T helper 1 (Th1) lymphocytes and later on cytotoxic lymphocytes (CTLs), monocytes, macrophages, natural killer cells (NKs), and dendritic cells (DCs), TIM-3 plays a key role in immunoregulation. Recently, a growing body of evidence has shown that its differential expression in various tumor types indicates a specific prognosis for cancer patients. Here, we discuss which types of cancer TIM-3 can serve as a prognostic factor and the influence of coexpressed immune checkpoint inhibitors, such as LAG-3, PD-1, and CTLA-4 on patients' outcomes. Currently, experimental medicine involving TIM-3 has significantly enhanced the anti-tumor effect and improved patient survival. In this work, we summarized clinical trials incorporating TIM-3 targeting monoclonal and bispecific antibodies in monotherapy and combination therapy and highlighted the emerging role of cell-based therapies.

## TIM-3 structure and function

T-cell immunoglobulin and mucin domain-containing protein 3 (TIM-3) is identified as an immune checkpoint that is expressed in various kinds of immune cells and plays a key role in immunoregulation. TIM-3 belongs to the TIM family, which includes three members in humans (TIM-1, TIM-3 and TIM-4) and eight members in mice (TIM-1 to TIM-8). Members of the TIM family are type I surface glycoproteins that share similar molecular structures containing a mucin domain, amino-terminal immunoglobulin variable domain (V domain) with five noncanonical cysteines, a transmembrane domain and a cytoplasmic tail [1, 2].

The IgV Tim-3 domain is formed of antiparallel beta sheets. A disulfide bond formed by four non-canonical cysteines binds the front and back sheets together to stabilize the IgV domain [3]. These two bonds form a unique "cleft" in Tim-3 and a small associated channel. The cleft formed by the CC′ and FG loops is stabilized by two non-canonical disulfide bonds, as well as a series of conserved ionic interactions and hydrogen bonds. It is a response site for ligand binding, such as phosphatidylserine. These unique conformational surface features may be involved in Tim-3's biological function of ligand recognition [4].

The IgV domain is stabilized by two hydrogen bonds, which are present in almost all IgSF domains—an interchain hydrogen bond formed by Trp-53 and a hydrogen bond formed by Tyr-109. There is also a salt bridge, which is a common feature of IgSF domains. TIM-3 contains the smallest mucin domain in the TIM family. This region contains an abundance of proline, serine, and threonine. The Tim protein enters the cell membrane through a tail made mostly of hydrophobic amino acids, which is embedded in the lipid layer and passes into the cell interior [5].

**Fig. 1:**
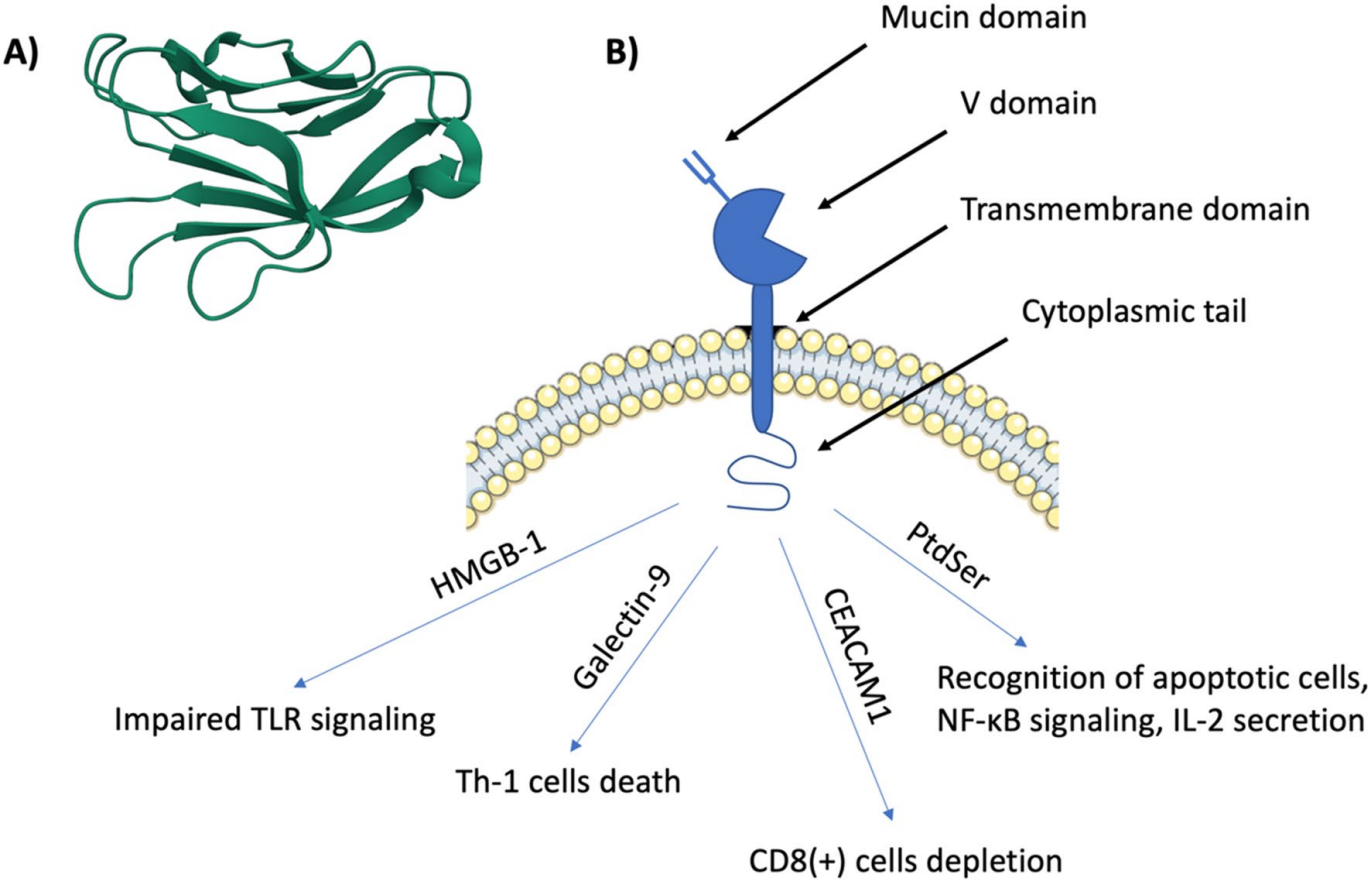
**a** Human TIM-3 N-terminal variable immunoglobulin (IgV) domain (PDB: 6DHB); **b** TIM-3 structure consists of mucin domain, V domain, transmembrane domain, and cytoplasmic tail. The binding of TIM-3 and HMGB-1 leads to impaired TLR signaling. Galectin-9 binding induces Th-1 cells death. CEACAM1 with TIM-3 interaction leads to CD8( +) cells depletion and finally, interaction with PtdSer enables the recognition of apoptotic cells, NF-kB signaling and IL-2 secretion

TIM-3 protein is involved in the regulation of the immune response and immune tolerance. It is expressed on Th1, Th17, monocytes, dendritic cells, and macrophages and is involved in CD8( +) T-cell depletion [6–9]. TIM-3 protein also plays a role in efferocytosis [5, 10, 11]. A key feature is its ability to bind specific ligands, which activate biochemical pathways. These include galectin-9 (Gal-9), cancer-embryonic antigen cell adhesion molecule 1 (CEACAM1), phosphatidylserine (PtdSer), high mobility group protein 1 (HMGB-1) [12, 13]. Figure 1 illustrates the structure and function of TIM-3 and its ligands.

Studies have shown that Galectin-9 bound to Tim-3 induces TH1 cell death by intracellular calcium flow, which induces apoptosis. Consequently, this causes induction of immune tolerance and suppression of TH1 and TH17 responses [12]. Carcinoembryonic antigen cell adhesion molecule 1 (CEACAM1) also induces immune tolerance, via heterodimer formation with TIM-3 [14]. They can bind in cis- and transforms, both of which regulate immune tolerance and can participate in CD8 + T cell depletion [15]. Another ligand of TIM-3 is phosphatidylserine (PtdSer), to which it binds via FG loops in the IgV domain. It is believed that the role of this binding may be related to the recognition of apoptotic cells [4, 16]. Furthermore, phosphatidylserine stimulates NF-κB signaling and IL-2 secretion [17].

The binding of TIM-3 by HMGB1 impairs nucleic acid binding to dendric cells endosomes, contributing to the inhibition of activation of the innate immune response [18]. Moreover, the binding of HMGB1 by TIM-3 results in the inhibition of immune response activation. TIM-3 competes with tumor cell-derived nucleic acids and binds HMGB-1. HMGB-1 bound to TIM-3 cannot participate in nucleic acid uptake into the cell, inhibiting TLR signaling activation in dendritic cells [18].

Bat3 is an adaptor protein that binds to the TIM-3 tail, preventing galectin-9-mediated T helper type 1 (TH1) cell death and promoting both proliferation and production of pro-inflammatory cytokines. Studies have shown that a lack of Bat3 expression results in impaired autoimmunity and accelerated tumor growth. It is associated with a decrease in the number of TH1 I TH17 cells and cytotoxic effector cells, while it increases the number of regulatory T cells and depletes CD8 + cells infiltrating the tumor [19, 20].

Tim-3 is a negative regulator of TLR-induced immune responses. It has been shown that overexpression of Tim-3 in macrophages significantly reduced TLR-induced production of pro-inflammatory cytokines. TLR4 is an important ligand for TIM-3, as Tim-3 negatively regulates the LPS/TLR4-induced pro-inflammatory response by inhibiting NF-κB activation [21]. Meanwhile, overexpression of TIM-3 on dendritic cells in the tumor area suppresses innate immune responses through recognition of nucleic acids by TLR receptors and cytosolic sensors in a galectin-9-independent mechanism [21, 22].

## TIM-3 expression

The T-cell immunoglobulin and mucin domain (TIM) gene family includes three genes in humans (TIM-1, TIM-3 and TIM-4 (T cell immunoglobulin and mucin-domain containing-4) [23]. Human TIM-3 is located on chromosome 5q33.3. It includes a large number of SNPs (single nucleotide polymorphisms), which affects the expression and activity of TIM-3 [24]. The analyses found that certain TIM-3 polymorphisms (−1516G/T, −574G/T, + 4259 T/G and four haplotypes) increase cancer risk in humans [24, 25]. TIM-3 was first identified on the surface of T helper 1 (Th1) cells, and afterward on cytotoxic lymphocytes (CTLs), monocytes, macrophages, natural killer cells (NKs) and also dendritic cells (DCs) [26, 27]. Expression of this molecule mainly involves IFNγ-producing CD4 + T cells, CD8 + T cells, NK cells, and FoxP3 + Treg cells [28]. However, it is also found on Th17 cells in reduced amounts than on Th1 cells [9]. TIM-3 expression on T cells depends on various inducers. The study by Anderson et al. indicates that the expression of this membrane protein on Th1 lymphocytes is regulated by the transcription factor T-bet, which binds to the Tim-3 promoter [29]. T-bet is a major Th1 transcription factor induced in inflammation by IL-12, leading to the expression of TIM-3. However, in immunomodulatory or exhausted states, IL-27 mediated by T-bet and NFIL3 is responsible for increasing TIM-3 transcription [28]. Another transcription factor is the signal transducer and activator of transcription 3 (STAT3). IL-10 and IL-27 together may be associated with the development of epigenetic changes at the *Havcr 2* locus [30]. IFN-b can induce TIM-3 production on the surface of Th1 cells in the absence of APCs [31]. Up-regulation of TIM-3 expression on CD4 + T cells is mediated by stimulation of the T cell receptor signaling pathway using antibodies against CD3 and CD28 or by chemicals such as forbol-micric acid (PMA) and ionophores [9].

Expression of Tim-3 at elevated levels on effector T cells is associated with the phenomenon of T-cell exhaustion during, e.g., viral infection, as well as negatively correlating with the proliferation and secretion of TNF-α and IFN-γ [27]. Carcinoembryonic antigen cell adhesion molecule 1 (CEACAM1) promotes TIM-3 expression in T cells [14]. Coexpression of these two molecules results in the up-regulation of TIM-3 expression on Jurkat T cells [32]. A study by Gautron et al. confirmed that TIM-3 expression on human Treg cells occurs after TCR stimulation. They also proved that TIM-3^+^ Treg cells suppress Th1, specifically Th17 cells, while TIM-3^−^ Treg cells inhibit only Th1. TIM-3^+^ Treg cells showed increased expression of FoxP3, lymphocyte-activation gene 3 (LAG-3), programmed death receptor-1 (PD-1), and cytotoxic T lymphocyte antigen-4 (CTLA-4) [33].

A study by Meggyes et al. demonstrates that in pregnant women, cytotoxic T cells, NK cells and subsets of NK cells with TIM-3 surface expression produce cytokines in an altered manner, which is reflected in cytotoxicity. Gal-9 levels in peripheral blood increase as pregnancy progresses, which has relevance to the interaction between Gal-9 and Tim-3 and the subsequent suppressive function [34]. Wang et al. observed that there is an increase in both the frequency of decidual CD8 + T (dCD8 + T) cells coexpressing TIM-3 and CTLA-4 in response to trophoblast, as well as cytokine production by this subgroup of lymphocytes [35]. It can be suggested that the pathways of these two molecules are significant in maintaining pregnancy. The PD-1 pathway is also important. PD-1^+^Tim-3^+^CD8^+^ T cells show higher expression of Th2-type cytokines than Tim-3^−^PD-1^−^CD8^+^ T cells. The Th2-type cytokines are involved in maternal–fetal tolerance [36].

The regulation of TIM-3 expression on CD8 + T cells also depends on a variety of factors. It has been investigated that IL-2, and especially IL-15, strongly induces the expression of this receptor [37]. Lake et al. noted that TIM-3 is mainly expressed on the plasma membrane rapidly after activation of effector CD8 + T cells, while it is located mostly intracellularly in the late stages of these cells [32]. This protein is recruited to the immune synapse upon CD8 + T cell activation, which has a significant role in regulating the immune response [38]. TIM-3 is also involved in differentiating human antigen-specific CD8 T cells via the mammalian target of rapamycin complex 1 (mTORC1). Evidence for this differentiation by activation of mTOR pathway is enhanced levels of phosphorylated S6 protein and *rhebl1* transcript [39].

High surface expression of TIM-3 on resting NK cells is increased upon activation. Gleason et al. examined that when comparing 2 subsets of CD56^Dim^ NK cells and CD56^Bright^ NK cells, greater resting *HAVCR2* gene expression characterizes the former. The expression of this receptor is induced by recombinant human IgG1 Fc multimer, monoclonal antibody (mAb) opsonized tumors or a number of interleukins (ILs) such as IL-2, IL-15, IL-12 and IL-18 [40–42]. Mentioned interleukins proportionally upregulate TIM-3 mRNA expression and T-bet mRNA expression. It can be assumed that the induction of NK and T cells is related to the same transcription factor. It was observed that during low concentrations of IL-12 and IL-18, the expression in CD56^Bright^ NK cells was markedly enhanced compared to CD56^Dim^ NK cells [40]. Studies indicate that NK cell function depends on the inducer of TIM-3 expression [42].

Soo-Jin Yoon et al. investigated that the induction of TIM-3 transcription on Jurkat T cells stimulated with anti-CD3 and anti-CD28 antibodies and in HMC-1 human mast cells treated with TGF-β is affected by mitogen-activated protein kinase (MAPK) activation [43]. Mast cells also express TIM-3 in a constitutive manner, which potentiates FcεRI-proximal signaling, leading to activation of these cells. The signaling pathways are largely similar to T cells [44]. The study by Jung Sik Kim et al. shows that TIM-3 transcript levels upregulate after TGF-β1 stimulation of HMC-1 cells. In addition, they localized TIM-3 promoter activity in mast cells, which includes a region from −349 to + 144 bp [45]. This transmembrane protein is also expressed on unstimulated peripheral blood CD14 + monocytes, however down-regulation occurs after TLR stimulation. This leads to the regulation of cytokine production, which affects the pro- and anti-inflammatory response of the immune system [46]. TIM-3 is also a regulator of macrophage function. Notably, it occurs in peritoneal exudative macrophages but has not been identified in peritoneal resident macrophages [5]. TIM-3 function is affected by cell type or expression level. Interestingly, CD8 + DCs have TIM-3 expression levels three times higher than CD8-DCs [16]. It should be emphasized that the mechanisms of regulation of TIM-3 expression on immune cells are still not thoroughly explored.

## TIM-3 in neoplasms

Expression of TIM-3 in a wide range of neoplasms has been a trending topic in recent years. The analysis of TIM-3 expression in the TCGA (The Cancer Genome Atlas, NIH, Center for Cancer Genomics) dataset indicated broad expression profiles in different types of cancer. Recently, a growing body of evidence has shown that its differential expression in various tumor types indicates a specific prognosis for patients [47–50]. Due to this, TIM-3 appears to have the potential to serve as a prognostic marker and valuable therapeutic target in solid tumors [51]. TIM-3 is currently of great interest due to its demonstrated efficacy in a number of preclinical cancer models, thus, it is important to understand its expression profile in tumors [52].

### Glioma

Gliomas are the most common type of malignant brain tumor [53]. The inflammatory microenvironment intensifies during glioma progression and promotes tumor growth and chemoresistance [54]. As the inflammatory microenvironment and immune escape are hallmarks of glioma progression, in many tumors, these mechanisms are interrelated and cooperate in the malignant transformation of cancer [55–57]. During glioma progression, tumor cells modulate the expression profile of immune checkpoint molecules and their ligands [58]. TIM-3 receptor modulates microglia function and regulates the interaction of microglia with nerve cells [59]. Recent studies revealed that TIM-3 is a representative immune checkpoint molecule significantly overexpressed in gliomas [60]. It was shown that TIM-3 expression is highly enriched in the phenotype of known malignant molecules, mostly in glioblastoma (the most common and aggressive type of brain tumor) and IDH-wildtype glioma [61]. Clinically, high expression of TIM-3 is an independent indicator of poor prognosis.

The activation of TIM-3 occurs primarily through its ligand Gal-9 [12]. The binding between Tim-3 and Gal-9 promotes tumor growth and suppresses the adaptive immune system [62]. A study by Simet al. found that the Tim-3/Gal-9 axis association with the NLRC4 inflammasome contributes to glioma development [58]. Tim-3/Gal-9 regulation was positively correlated with NLRC4 inflammasome, NLRC4, and caspase-1 expression. What is more, Tim-3/Gal-9 expression was strongly positively correlated with caspase-1 activity as it induced programmed cell death in glioma cells. A protein–protein interaction analysis proved that the FYN-JAK1-ZNF384 pathways are bridges in regulating the NLRC4 inflammasome by Tim-3/Gal-9 pathway. Another study revealed that Tim-3 might be specifically upregulated on microglia by stimulation of adenosine 50-triphosphate disodium salt (ATP), which is released from damaged neural cells or lipopolysaccharide (LPS)—the bacterial endotoxin [63]. These data suggest that the level of Tim-3 expression on microglia is altered by pathological factors, confirming that Tim-3 plays an important role in response to environmental factors. The same study showed that Tim-3 stimulation triggers microglia into an anti-inflammatory state. Antibody-mediated activation of TIM-3 increases the expression of TGF-β, TNF-α, and IL-1β in microglia. Furthermore, upregulation of Tim-3 may increase the interaction between Tim-3 and Gal-9 and activate the Toll-like receptor 4 (TLR-4) pathway [64].

In glioblastoma, which shares signaling pathways between glioma and immune cells, TIM-3 is one of tumor and non-tumor cells' most common co-inhibitory immune checkpoints [64]. Glioma cell-intrinsic TIM-3 is involved in inducing macrophage migration and transition to anti-inflammatory/pro-tumorigenic phenotype by a TIM-3/interleukin 6 (IL6) signal. In mechanism, as one of the key regulators of IL6, TIM-3 regulates its expression through NF-κB activation. An in vivo study by Guo et al. proved that inhibiting this loop by antibodies prolonged the survival of tumor-bearing mice [65]. In a similar study in glioblastoma models, the blockade of Gal-9/Tim-3 signaling inhibited M2 macrophage polarization and suppressed tumor growth [66]. In advanced solid tumors, it is widely thought that macrophages polarized toward the M2-like phenotype are associated with tumor progression and suppression of tumor-specific immunity [67]. Thus, targeting TIM-3 may provide a new therapeutic opportunity for glioblastoma. Taken together, these data support the hypothesis that modulation of TIM-3 signaling may have therapeutic value in treating a range of clinical disorders, from autoimmunity to cancer.

#### Thyroid cancer

TIM-3 and its ligand galectin-9 expression differ in thyroid cancer than in normal thyroid tissues [68]. The expression profile varies significantly in different pathological stages. In papillary thyroid carcinoma (PTC), which is the most common form of well-differentiated thyroid cancer, most checkpoint molecules, including LAG-3, PD-1, ICOS, and IDO1, are significantly reduced compared to TIM-3, whose expression is significantly enriched [69]. In lymph node metastasis, overexpression of TIM-3 is associated with the coexpression of PD-L2, TIGIT, ICOS, PD-L1, and CD27 checkpoints. In samples with the BRAFV600E mutation, TIM-3 is overexpressed, as well as both PD-L2, TIGIT, ICOS, PD-L1, and LAG3, while CD27 is only repressed. Merely in radiation-induced PTC, TIM-3, and CD27 are downregulated compared to non-radiation-treated ECR. It was shown that in patients with regionally metastatic differentiated thyroid cancer, PD-1 + Tim-3 + CD8 + T cells are variably deficient in their ability to produce IL2, TNFα, and IFNγ [70]. Moreover, even though PD-1 + Tim-3 + CD8 + T cells can degranulate, their cytotoxic potential may be reduced.

In medullary thyroid carcinomas, expression of TIM-3, CTLA-4, and coexpression of PD-1/PD-L1 are related to poorer structural recurrence-free survival [71]. In thyroid tumors, a significant effect was observed on tumor-associated macrophages (TAM), where induction of their activation toward a pro-cancer phenotype was observed along with elevation of the checkpoint marker TIM-3 [72]. It was confirmed that TIM-3 induces tumor-promoting M2-like macrophage polarization [70]. Interestingly, experiments using TIM-3-blocking antibodies partly reversed these effects, suggesting a role for this receptor in the activation and pro-tumorigenic effects of TAMs in thyroid cancer in vitro [72]. In the study by Pani et al., analysis performed within the thyroidal immune infiltrate revealed that anti-TIM-3 treatment was associated with a significant change in the expression of 9167 genes compared to animals treated with isotype control [73]. Another study showed that PD-1 and Tim-3 blocking could effectively enhance the NK function in ATC patients. Interestingly, PD-1 and Tim-3 blockade was effective at reinvigorating both the more impaired NK cells (CD56hiCD16hi/lo) and the less impaired NK cells (CD56loCD16hi) [74].

#### Lung cancer

In patients with lung cancer, TIM-3 is characterized by a relatively high positive expression rate on both CD4( +) and CD8( +) TILs from human lung cancer tissues [75, 76]. Significantly, TIM-3 expression on CD4 + T cells correlates with nodal metastasis and advanced cancer stage [76]. Thus, patients with TIM-3-positive tumor cells present a significantly shorter survival time than patients with TIM-3-negative tumors [77]. TIM-3 expression in non-small cell lung cancers (NSCLC) tumor cells is related to the histologic type and pathologic T classification of the disease [50]. Some studies have shown that TIM-3 is detected on tumor cells in 86.7% of patients with primary NSCLC [50]. The expression profile of inhibitory coreceptors on tumor-infiltrating T cells from patients with NSCLC shows a clear correlation between PD-1, Tim-3, CTLA-4, LAG-3, and BTLA expression on intratumoral CD8( +) T cells [78, 79]. The correlation between increased expression of these inhibitory coreceptors is associated with the progressively impaired ability of T cells to respond to polyclonal activation and disease progression [80]. Interestingly, in EGFR-mutant adenocarcinomas, expression of TIM-3, PD-1, and LAG-3 is lower and shows limited association with tumor mutational burden [80]. TIM-3 is overexpressed in NK cells and macrophages, while PD-1 and LAG-3 are mainly localized on T/NKT cells. Co-expression of PD-1, LAG-3, and TIM-3 has been linked to marked T-cell activation (CD69/CD137), effector function (Granzyme-B), and proliferation (Ki-67), but also enhanced levels of proapoptotic markers (FAS/BIM). A recent study revealed that PD-1 interacts with Gal-9 and TIM-3 to attenuate Gal-9/TIM-3-induced apoptosis of PD-1 + TIM-3 + T cells in cancers and demonstrates that Gal-9 is upregulated by the inflammatory cytokines IFNβ and γ [81]. The potent inhibition of human Gal-9 by neutralizing antibodies may open new avenues for lung cancer immunotherapy [81–83].

#### Colorectal cancer

Colorectal carcinoma is a malignant neoplasm that is closely related to inflammation [84]. Immune dysfunction with T-cell exhaustion has been postulated as the main cause, where CD4 + and CD8 + T-cells residing in tumor tissues express TIM-3 and PD-1 on their surface [85, 86]. As CD8 + T cells migrate to tumor sites to eliminate tumor cells [87], an increase in the number of tumor-infiltrating CD8 + T cells in the tumor microenvironment correlates with better clinical outcomes in human cancers [88–91]. Tumor-infiltrating CD8 + T cells express co-inhibitory molecules such as TIM-3. In colorectal cancer, a key role in immune regulation is played by both TIM-3 and its three ligands—galectin 9 (Gal9), HMGB1, and CEACAM1 [92]. Since an increase in the number of tumor-infiltrating CD8 + T cells improves the clinical outcome of human colorectal cancer, the removal of tumor-infiltrating T cells with apoptosis may cause immune dysfunction [93–95]. Kang et al. have shown that the percentage of TIM-3 + cells in tumor-resident tissues is significantly higher compared to CD8 + T cells from peripheral blood in the same patients [96]. When compared to the TIM-3- population, TIM-3 + CD8 + T cells secrete more effector cytokines such as IIFN-γ, TNF-α, and IL-2. The number of apoptotic cells is elevated in CD8 + T cells infiltrating the tumor compared to TIM-3 + cells in the spleen. Tumor cells secrete Gal-9, which, in interaction with TIM-3 on infiltrating CD8 + T cells, induces apoptosis in functionally active, tumor-infiltrating TIM-3 + CD8 + T cells.

Tim-3 expression levels affect tumor size, tumor node metastasis staging, and distant metastasis [47]. Moreover, TIM-3 is co-expressed and forms a heterodimer with its ligand CEACAM1 [97]. The presence of CEACAM1 confers an inhibitory function to TIM-3. CEACAM1 promotes the expression of TIM-3 on the cell surface and maturation, thus T cells lacking CEACAM1 are hyperactive, with reduced expression of TIM-3 and regulatory cytokines on the cell surface. It was shown that coexpression is correlated with advanced stage and could be an independent risk factor for colorectal cancer [98]. Importantly, in vivo studies revealed that co-blockade of CEACAM1 and TIM-3 leads to enhanced anti-tumor immune responses with improved elimination of colon cancer tumors [97].

#### Head and neck cancer

Head and neck cancer (HNC) is a heterogeneous group of malignancies. HNC often occurs in the upper neck, such as the tongue, pharynx, nasopharynx and lip. The most common histological subtype is referred to as head and neck squamous cell carcinoma (HNSCC), which is mainly associated with tobacco and alcohol consumption [99].

In HNSCC, TIM-3 expression has been observed in inflammatory cells, especially CD8 + T cells and MDSCs [100]. This expression is elevated compared to normal mucosa and dysplasia. Enhanced expression of TIM-3 is closely related to tumor size, and recurrence, lymph node metastasis and stage. In contrast, there is no association with gender, age, tumor location, degree of tumor differentiation, or history of smoking or alcohol consumption [101]. Both the expression of TIM-3 and its ligand Gal-9 are upregulated not only on tumor cells but also on immune cells such as Tregs and M2 macrophages in the tumor stroma [102]. When TIM-3 is coexpressed with CEACAM1 or PD-1, T-cell depletion is observable [103]. T cells lacking CEACAM1 are hyperinflammatory with reduced expression of cell surface TIM-3 and regulatory cytokines, but this effect is reversed when T cells specifically express CEACAM1 [97]. Thus, TIM-3 and CEACAM1 form an axis that can inhibit immune responses and thereby reduce their anti-tumor immunity. Correlated PD-1 and TIM-3 antigens reflect the immune status of CD8 + T cells in the tumor microenvironment. A study by Jie et al. showed that during cetuximab therapy, an increased population of CD8 + TILs co-occurred with granzyme B/perforin and PD-1/TIM-3, suggesting a regulatory role for these checkpoint receptors in promoting cytolytic activity [104].

In vivo studies using anti-TIM-3 monoclonal antibodies have shown that it is possible to effectively inhibit tumor growth by restoring effector T cell function and reducing the recruitment of myeloid-derived suppressor cells to tumor microenvironment in a CXCL1-dependent manner [105]. A study in mouse orthotopic models of HNSCC showed that Anti-TIM-3 treatment concomitantly with anti-PD-L1 and radiotherapy led to a significant delay in tumor growth, increased T-cell cytotoxicity, reduced Tregs, and improved survival [106]. However, this effect was not permanent. The solution was found to be targeted Treg depletion, which restored anti-tumor immunity in mice treated with RT and dual immune checkpoint blockade and induced tumor rejection and induction of immune memory. Interestingly, the same study also showed that in tumors treated with radiotherapy and anti-PD-L1 triggered overexpression of TIM-3 on CD8 T cells and Tregs.

#### Gastric cancer

In gastric cancer, TIM-3 expression is positively correlated with a worse prognosis [48, 107]. Interestingly, TIM-3 expression is significantly lower in the tumor mucosa than in the mucosa of controls [107, 108]. However, it increases markedly in patients with lymphovascular invasion. It was noted that the expression profile significantly impacts patient survival—high Gal-9 expression and low Tim-3 expression were particularly associated with longer overall survival. However, a study by Wang et al. demonstrated that a high level of Gal-9 was negatively correlated with poor prognosis [48]. The role of Gal-9 is therefore controversial, and the discrepancy may be due to its different functions in different immune states of patients [109]. Since the function and tumorogenic role of gal-9 in gastric cancer remains poorly understood, it is TIM-3 that is thought to be a relatively promising biomarker and therapeutic target.

Tim-3 plays an important role in the development and progression of gastric cancer, while its level of expression on CD4 + T cells influences clinicopathological parameters such as tumor size, lymph node metastasis, and depth of tumor invasion [108]. CD8 + T cells positive for both Tim-3 and for PD-1 produce significantly less IFN-γ, TNF-α, IL-2, and the increased number of these cells is closely associated with impaired CD8 + T cell function in gastric cancer patients [110]. In contrast, CD8 + T-cell dysfunction is associated with impaired response to chemotherapies and worse disease-free survival [111]. The study by Chen et al. found that subgroups of tumor-infiltrating TIM-3 + cells predicted poorer therapeutic responsiveness to fluorouracil-based adjuvant chemotherapy [112]. Tim-3 expression by monocytes/macrophages may also be an important mechanism in gastric cancer progression, as Gal-9/Tim-3 signaling can significantly stimulate monocytes to secrete IL-6, IL-8, and IL-10, the expression of which in the tumor microenvironment is strongly associated with poor treatment outcome [113, 114]. Clinical analyses have shown that NK cells obtained from gastric cancer patients also exhibit significantly higher levels of TIM-3 than healthy control cells, which is associated with the advanced tumor stage [115]. In studies conducted in a tumor-bearing mouse model, Tim-3 levels in NK cells increased with tumor growth, indicating that tumor progression can induce Tim-3 expression in NK cells [115].

#### Liver cancer

In liver cancer, TIM-3 is an independent indicator of poor prognosis and may play an essential role in the progression, invasion, and metastasis [116]. Elevated TIM-3 expression occurs substantially on CD4 + and CD8 + T cells infiltrating tumor tissues compared to cells infiltrating adjacent tissues [117]. It was shown that hepatocyte-specific Tim-3 overexpression enhances tumor cell growth through IL-6 autosecretion, and it increases the metastasis-forming ability of HCC cells by promoting the epithelial-mesenchymal transition [118]. Furthermore, this expression is also induced on immune cells as a result of chronic stimulation and the cytokine environment (IL-4, TGF-β, and IL-6) in the tumor microenvironment [118]. Expression levels of TIM-3 are dependent on disease severity and positively correlated with aspartate aminotransferase (AST), alanine aminotransferase (ALT), total bilirubin (TB), and international normalized ratio (INR) [13]. T cells expressing TIM-3 are highly coupled to PD-1 and exhibit the lowest levels of granzyme B, IFN-γ, and TNF-α, implicating a suppressive role for these immune checkpoints in liver cancer [119].

The cause of most cases of cirrhosis and primary liver cancer is hepatitis B (HBV) infections, which are responsible for immune system dysfunction [120]. In patients with HBV, there is increased expression of Tim-3 on circulating monocytes, which activates the inflammatory response through the promotion of inflammatory cytokine production and Th17 responses [121]. In addition, patients with chronic HBV infection have been observed to have elevated Tim-3 expression in many other immune cell types, such as cytotoxic T cells, T helper cells, dendritic cells, macrophages, and natural killer cells [1]. Since increased Tim-3 expression inhibits the antiviral immune response, Tim-3 may be a potential target to control infection in HBV patients [122]. Indeed, recent studies have shown that blocking Tim-3 signaling with anti-Tim-3 antibodies results in markedly improved proliferation and release of antiviral cytokines by CD8 + T cells in response to HBV-specific antigenic peptides [123]. Another study proved that Tim-3 blockade promotes α-Galcer-induced inhibition of HBV replication by invariant NKT cells [124]. Dual blockade of Tim-3 plus PD-1 also has shown positive results in that they enable inhibition of hepatocellular carcinoma cell growth into senescence, suggesting that their modulation may be a rational target for new immunotherapeutic approaches [125].

#### Pancreatic cancer

Tim-3 expression in pancreatic cancer tissues has been observed to be substantially higher compared to peri-pancreatic and normal tissues. In contrast, no statistically significant difference was observed between Tim-3 expression in peri-pancreatic and normal tissues [126]. It was demonstrated that the amount of Tim-3 expression in pancreatic cancer is influenced by smoking, fasting, blood glucose levels, tumor size, and TNM stage. Features that did not affect changes in expression appeared to be gender, age, tumor location, pathologic type, or degree of tumor differentiation.

Interestingly, the study by Nakayama et al. found that TIM-3 had no statistical association with patient survival [127]. The investigation showed that the ratio of PD-1 expression to CD8 + T cells was more significant in prognosis. Accordingly, no correlation was observed between the Tim-3/Galectin-9 and CD155/TIGIT pathways and patient prognosis. However, a study by Bai et al. showed that after blocking PD-1 and TIM-3, CD8 + T-cell functions improved, thereby increasing disease-free survival in pancreatic cancer patients after tumor resection. This study identified that CD8 + CXCR5 + T cells were a potent subset of CD8 + T cells that were highly enriched in pancreatic cancer patients and could respond to anti-PD-1/anti-TIM-3 blockade by further regulating function [128]. To date, there are still few studies involving TIM-3 expression in pancreatic cancers, making the topic controversial. The mechanisms involved in the TIM-3-mediated immune response have not been fully explored.

#### Breast cancer

Since the currently available immune checkpoint inhibitors seem to benefit only a small number of women with breast cancer, the great hope is TIM-3, which is a new target for immunotherapy [129]. Interestingly, contrary to previously discussed cancers, the presence of TIM-3 on TILs in breast cancer is an independent favorable prognostic factor [130, 131]. Unfortunately, the mechanism underlying this effect is not yet known.

In early breast tumors, TIM-3 expression is correlated with improved breast cancer-specific survival [130]. These findings were also supported in a study on triple-negative breast cancer, where TIM-3 was associated with improved durability, despite its association with poor clinical and pathologic features [132]. In invasive ductal breast cancer (IDC), the observations were similar in the fact that TIM-3 + /CD8 + T cells were correlated with lymph node metastasis, histologic grade [133]. TIM-3 expression is found in all infiltrating cells present in breast cancer and correlates with other immune checkpoint inhibitors such as LAG-3, CTLA-4, and PD-1, which may underscore the importance of their interaction in the microenvironment [131, 134]. It was shown that TIM-3 exhibits strong overexpression on CD8 + T cells, T cells (general), B cells, monocytes, and TAM. In tumors with TIM-3 overexpression correlated with CTLA-4 and LAG-3, high levels of infiltrating NK cells and DCs were observed, indicating the regulatory capacity of immune checkpoints in the infiltration of these cells.

In vitro study conducted by Saleh et al. revealed that PD-1 and PD-L1 co-blockade additionally elevates TIM-3 and LAG-3 coexpression on CD4 + CD25 + T cells, including Tregs [135]. However, patients with high levels of TIM-3 treated with adjuvant chemotherapy had more favorable survival than those whose expression was lower [131]. TIM-3 expression by intratumoral CD103 + dendric cells has been proven to regulate chemokine expression during paclitaxel treatment, and by administering an anti-TIM-3 antibody, it is possible to increase granzyme B expression by CD8 + T cells and effectuate the immune response to chemotherapy [136]. I another study, it was shown that patients in advanced TNBC with elevated plasma Tim-3 or CTLA-4 expression responded more to treatment with camrelizumab (an anti-PD-1 immune checkpoint inhibitor) with lapatinib (a vascular endothelial growth factor receptor-2 inhibitor) [137].

#### Renal cancer

Analysis using the TCGA database pointed to a higher frequency of *TIM-3* gene amplification in Renal cell carcinoma (RCC) compared to other cancer types. The reason may be that the *TIM-3* gene is located on chromosome 5q, which undergoes frequent amplification in RCC [138]. RCC is a heterogeneous tumor in which immune checkpoints are differentially expressed between primary and metastatic tumors [139]. Multivariate analysis showed that TIM-3 expression in metastases is significantly higher than in primary tumors and may be more indicative of patients' prognosis. Therefore, Zhang et al. suggest that more attention should be given to evaluating metastatic sites in clinical practice and research to improve the efficacy of mRCC immunotherapy [140]. High TIM-3 expression is a factor directly associated with poor prognosis in ccRCC, affecting cancer-specific survival as well as progression-free survival [141]. Furthermore, TIM-3 undergoes differential expression between sunitinib-resistant and sensitive groups and is associated with the benefit of sunitinib treatment in patients with metastatic renal cell carcinoma [142]. In patients with upregulated Tim-3 expression, a higher frequency of IL-10-producing tumor-infiltrating B cells is widespread [143]. Besides, the tumor-infiltrating B-cell supernatant has been proven to suppress the inflammation of autologous blood T cells, indicating the existence of immunosuppressive activity of tumor-infiltrating B cells. Elevated Tim-3 levels on both TIL CD4 + T cells and TIL CD8 + T cells are associated with higher stages of cancer [144]. Further, analysis of the mechanism of CD8 + TIL Tim-3 cells showed impairment of the Stat5 and p38 signaling pathways. Blocking the Tim-3 pathway restores cell proliferation and increases IFN-γ production in TIL CD4 + and CD8 + T RCC cells.

Recent studies have shown that disrupting Tim-3 expression in clear cell renal cell carcinoma can reduce cancer invasion by enhancing anoikis (a form of programmed cell death), identifying Tim-3 as a potential therapeutic target [145]. In vivo, suppression of RCC tumor growth was observed by anti-TIM-3 mAb treatment. Further, in the group receiving additive monocytes, the treatment reduced the infiltration of M2 macrophages expressing CD163, suggesting that TIM-3 may promote the differentiation of protumor myeloid cells in the tumor microenvironment [146].

Kato et al., in their study, presented that TIM-3 expression on cancer cells could be a potential predictor of the efficacy of anti-PD-1 therapy [147]. However, they emphasize that TIM-3 alone may not be sufficient to predict efficacy, but simultaneous evaluation of both TIM-3 and its ligand, HMGB1, may be sufficient. This is because HMGB1 is a necrosis-associated ligand, and HMGB1 release from the nucleus was only found in the area of tumor necrosis after anti-PD-1 therapy.

#### Melanoma

Tim-3 expression is elevated on the surface of NK cells, monocytes, and mDCs in melanoma patients and healthy donors [148]. Importantly, there is no significant difference in the Tim-3 expression profile across these cells between healthy donors and melanoma patients. A study by Fourcade et al. found that Tim-3 expression is elevated on tumor-induced NY-ESO-1-specific CD8 + T cells of patients with advanced-stage melanoma [148]. Moreover, the vast majority of Tim-3 + CD8 + T cells specific for NY-ESO-1, upregulate PD-1 expression, and the Tim-3 + PD-1 + NY-ESO-1-specific CD8 + T cell subtype represents a highly dysfunctional tumor-induced T cell population.

In the surrounding melanoma mast cells, expressions of GAL-9 and TIM-3 are highly elevated [149]. Interestingly, it was shown that in the tumor environment, mast cells express GAL-9 at levels about 1000 times higher than melanoma cells. This dependence may result in reduced adhesion properties of tumor cells. Also, it has been proven that upregulation of TIM-3 by TGF-βI can inhibit the local immune response against tumor cells. In endothelial cells, Tim-3 may be expressed after stimulation with its ligand TLR4, which is released by cancer cells [150, 151]. The interaction of Tim-3 expressed by endothelial cells with the non-galectin-9 receptor on melanoma cells activates a distinct signaling pathway. This results in activation of NF-κB, which promotes cell proliferation and increases resistance to apoptosis through the upregulation of Bcl-2 and Bcl-xL and the downregulation of Bax proteins. These interactions' effects include increased survival of melanoma cells in the bloodstream, arrested in the lungs, and more metastatic nodules.

The study by Silva et al. demonstrated that NK cells from patients with metastatic melanoma were functionally impaired, and TIM-3 expression correlated with disease stage and poor prognostic clinical outcomes [152]. Tim-3 blockade reversed this depleted phenotype and improved their cytotoxicity. Combination therapy with Anti-Gal-9 did not have the same effect, suggesting other natural Tim-3 ligands may be involved. In an in vivo study, Ab-mediated blockade of Tim-3 inhibited the growth of immunogenic mouse melanomas in hosts with T-competent cells [153]. However, administration of Tim-3 Ab in T-cell-deficient mice induced tumorigenesis of highly and less immunogenic mouse and human melanomas. Thus, it was noted that melanoma-Tim-3 activation inhibited, while its blockade increased, the phosphorylation of proliferative mediators of MAPK signaling. Importantly, pharmacological inhibition of MAPK allowed Tim-3 Ab-induced tumorigenesis to be reversed in T-cell-deficient mice, enhancing anti-tumor activity. These results demonstrate that targeting MAPKs may be a key combination strategy to circumvent the adverse implications of melanoma treatment.

## Anti-TIM-3 antibody-based therapies

Dysregulated Tim-3 expression is associated with immune exhaustion in cancer and viral infections. The mechanism by which Tim-3 mediates inhibitory signaling remains unclear, and several mechanisms are suggested that Tim-3 may promote tumor progression, including facilitating tumor cell migration and invasion, activation of the IL-6-STAT3 pathway leading to direct suppression of CD4 + T cells to inhibit Th1 polarization or activation of mTOR function in AML cells. TIM-3 can regulate both the innate and adaptive immune response due to its inhibitory function of Treg and the function of myeloid-derived suppressor cells, contributing to the improvement of the immune response. Blocking Tim3 with mAb increases T-cell proliferation and immune function [154].

A newly developed Tim-3 antibody with therapeutic potential against Tim-3-associated immune disorders was studied by investigating its effects on PBMCs, T cells, and monocytes/macrophages. The new monoclonal antibody (L3G) against human Tim-3 increases STAT1 phosphorylation in both T cells and monocytes/macrophages and enhances IL-2 and type I interferon expression; in addition, L3G inhibited H1N1 viral infection in immune cells, as shown by Ge Li et al. [155].

The study by Koyama et al. observed, in a mouse model of lung adenocarcinoma, an upregulation of alternative immune checkpoints, particularly TIM-3, in T cells bound to the PD-1 antibody and demonstrated a survival advantage when a TIM-3 blocking antibody was added after PD-1 blockade failed. These data suggest that the upregulation of TIM-3 and other immune checkpoints may be target biomarkers associated with adaptive resistance to PD-1 blockade [156].

However, monoclonal therapy has no spectacular therapeutic effect and mechanism, as confirmed in the testing of anti-mouse mAb TIM-3 activity against experimental and carcinogen-induced tumors. However, comparative and combination studies of anti-TIM-3 with anti-CTLA-4 and anti-PD-1 against experimental and induced tumorigenesis suggested that these agents could be well tolerated and highly effective in combination [157]. Therefore, blockade of co-inhibitory receptors has emerged as an effective treatment option for many human cancers. Monoclonal TIM-3 antibodies in clinical trials inhibit only some, but not all, ligands. We can distinguish PD-1, CTLA-4, TIM-3, LAG-3, and TIGIT as Co-inhibitory receptors by their important role in activated T cells, regulatory T cells, and exhausted T cells. These receptors suppress T cell function in the tumor microenvironment, causing T cell dysfunction [158].

As mentioned, the increased production of Galectin-9 (Gal9) molecules induces the binding of Tim-3 molecules expressed on Tim-3 expressing effector CD8 + T cells in the tumor microenvironment, which lead to apoptosis of effector T cells. This results in the ligation of PDL1 with PD1 (programmed cell death protein 1) and galectin9 with Tim-3 molecules, causing suppression of anti-tumor immunity due to the downregulation of T-cell function, essentially creating a negative feedback loop. Monotherapy produces a partial response, while combined therapy with anti-PD-1 and Tim-3 may effectively achieve a complete response [159]. Ongoing clinical trials have already confirmed the safety and efficacy of combination therapy. The Curigliano study enrolled 133 patients treated with sabatolimab and 86 patients on sabatomimab in combination with spartalizumab [160]. Sabatolimab is mAb that binds TIM-3 with subnanomolar affinity, blocks interaction with its ligand, phosphatidylserine, and partially blocks the interaction of TIM-3 with Galectin-9 and Spartalizumab blocks interaction with programmed death-ligand 1/2 by binding PD-1 with subnanomolar activity. The results supported that dual blockade of TIM-3 and PD-1 is more effective than targeting each of these pathways separately, as no response was observed in patients receiving a single sabatolimab.

In preclinical studies in mice with solid tumors, CD8 + TILs co-expressing TIM-3 and PD-1 show a profound defect in T cell effector function. The combined targeting of these pathways was shown to be highly effective in controlling tumor growth and restoring IFN production by T cells [129, 161].

Bispecific Abs, which can simultaneously target two oncogenic antigens or epitopes, show promising efficacy. They are mainly divided into two subtypes—the first includes those with an Fc region, while the second lacks an Fc region. In the first category, the IgG-like antibody, with Fc-mediated effector functions, binds the tumour antigen, forming a membrane attack complex, resulting in tumour lysis. The second alters biological responses and may trigger apoptosis by inducing this effect solely on the binding of Ab to the tumor antigen [129]. The successfully developed bispecific antibodies BP1210 and BP1212 against TIM-3 and CD39, negative regulators of anti-tumor immunity, overcome immunosuppression in the tumor microenvironment. Expression of TIM-3 and CD39 is simultaneously induced in deprived T cells and DCs and inhibits the antitumor activity of T cells and DCs. Anti-tumor immunity is synergistically enhanced by inhibiting CD39 in combination with TIM-3 blockade [162].

**Fig. 2:**
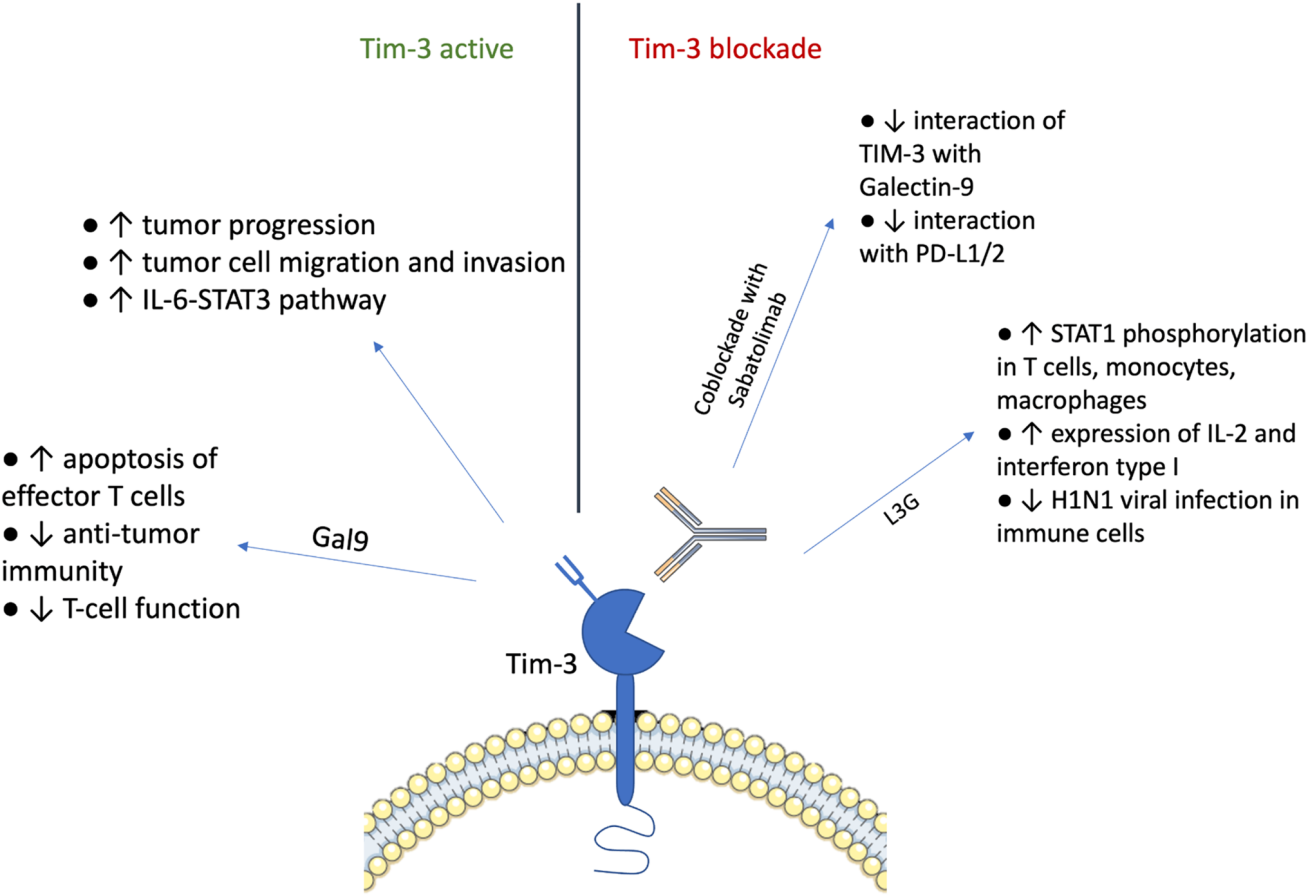
Schematic representation of TIM-3-mediated immunosuppressive signaling

Furthermore, in the research of Jing Chen et al. effect of dual immune checkpoint blockade and its potential in targeted cancer immunotherapy is based on the recombinant sP1T3 fusion protein. With PD-1 and TIM-3 ligands presented on the cancer cell surface, a recombinant fusion protein is connected and successfully constructed that can bind to both. Designated as sP1T3, indicates specific binding to these ligand-expressing cancer cells, which is confirmed by comparison with negative control using cell lysate without sP1T3 [163]. Figure 2 shows a graphical representation highlighting the TIM-3-associated pathway of immunosuppressive signaling.

## Experimental medicine involving TIM-3—clinical trials

A number of clinical trials regarding anti-TIM-3 antibodies have been conducted up to date. Therapies consist of monoclonal antibodies both in monotherapy and combination treatment and bispecific, the majority of which are anti-TIM-3/anti-PD-1 antibodies. According to clinicaltrials.gov, there are currently 110 study trials for TIM-3-related drugs.

### Anti-TIM-3 monoclonal antibodies

The majority of assessed treatments are combination therapies of monoclonal anti-TIM-3 antibodies with anti-LAG-3 and anti-PD-1 antibodies. Studies show that targeting TIM-3 and PD-1 simultaneously is more effective than targeting either pathway alone. The synergistic effect of the PD-1/TIM-3 coblockade was found in preclinical cancer models [52]. Sabatolimab (MBG453) is a monoclonal, T-cell immunoglobulin domain and mucin domain-3 (TIM-3) binding antibody. Phase I/II clinical trial assessing the safety and effectiveness of sabatolimab as single-agent and in combination with spartalizumab (anti-PD-1 mAb) in patients with advanced solid tumors. Therapy was well tolerated. Furthermore, sabatolimab monotherapy was not effective, while patients receiving combination treatment showed preliminary signs of antitumor activity [164]. Furthermore, anti-TIM-3 antibodies, including MBG453, combined with other checkpoint inhibitors, can be a promising path in leukemia immunotherapy [165]. The examples of clinical trials involving anti-TIM-3 monoclonal antibodies in monotherapy and combined therapy are shown in Table 1.

**Table 1:** Examples of Clinical Trials involving Anti-TIM-3 monoclonal antibodies

Identifier	Patients number	Recruitment Status	Condition or disease	Target	Therapy protocol	Short description
NCT03489343 [166]	24	Completed	Metastatic cancer Solid tumor Lymphoma	TIM-3	Drug: Sym023	Phase I study of Sym023 (Anti-TIM-3) for patients with locally advanced/unresectable or metastatic solid tumor malignancies or lymphomas
NCT04823624 [167]	20	Not yet recruiting	Myelodysplastic syndromes	TIM-3	Drug: MBG453	Phase II study of MBG-453, a humanized monoclonal antibody, in treating myelodysplastic syndromes (MDS)
NCT04623892 [168]	50	Unknown	Advanced solid tumors	TIM-3	Drug: TQB2618 injection	Phase I study of TQB2618 (Anti-TIM-3) antibody in monotherapy
NCT05287113 [169]	162	Recruiting	Head and neck cancer	TIM-3 LAG-3	Drug: Retifanlimab Drug: INCAGN02385 Drug: INCAGN02390 Drug: Placebo	Retifanlimab in Combination With INCAGN02385 (Anti-LAG-3) and INCAGN02390 (Anti-TIM-3) as First-Line Treatment in Participants With PD-L1-Positive (CPS ≥ 1) Recurrent/Metastatic Squamous Cell Carcinoma of the Head and Neck
NCT04641871 [170]	148	Active, not recruiting	Metastatic cancer Solid tumor	TIM-3PD-1 LAG-3	Drug: Sym021 Drug: Sym022 Drug: Sym023 Drug: Irinotecan Hydrochloride	Phase I study of 3 combinations: Sym021 (Anti-PD-1) and Sym022 (Anti-LAG-3), Sym021 and Sym023 (Anti-TIM-3), Sym021 + Sym023 + irinotecan in patients with biliary tract carcinomas and with esophageal squamous cell carcinoma
NCT04139902 [171]	56	Recruiting	Melanoma stage III and IV	TIM-3PI-1 PD-1	Drug: Dostarlimab (TSR-042) (singly) Drug: Dostarlimab (TSR-042) and TSR-022 (combination)	Anti-PI-1 inhibitor (TSR-042) or anti-PD-1/anti-TIM-3 combination (TSR-042 / TSR-022) in patients with operable melanoma
NCT03680508 [172]	42	Recruiting	Adult primary liver cancer Advanced adult primary liver cancer Localized unresectable adult primary liver cancer	TIM-3 PD-1	Drug: TSR-022 and TSR-042	Combination of TSR-022 (cobolimab, TIM-3 binding antibody) and TSR-042 (dostarlimab, PD-1 binding antibody) in treating patients with locally advanced or metastatic liver cancer
NCT03099109 [173]	275	Active, not recruiting	Solid tumor	TIM-3 PD-L1	Drug: LY3321367 Drug: LY3300054	Phase I study of LY3321367 (Anti-TIM-3) administered alone or in combination with LY3300054 (Anti-PD-L1) in participants with advanced relapsed/refractory solid tumors
NCT04370704 [174]	146	Recruiting	Melanoma	TIM-3PD-1 LAG-3	Drug: INCAGN02385 Drug: INCAGN02390 Drug: INCMGA00012	This phase I study of the following combinations of study drugs: INCAGN02385 (Anti-LAG-3) + INCAGN02390 (Anti-TIM-3) and INCAGN02385 (Anti-LAG-3) + INCAGN02390 (Anti-TIM-3) + INCMGA00012 (Anti-PD-1)
NCT05645315 [175]	127	Recruiting	Advanced solid tumor	TIM-3 PD-L1	Drug: TQB2618 injection and TQB2450 injection	TQB2618 (Anti-TIM-3) injection combined with TQB2450 (Anti-PD-L1) injection in patients with advanced malignant solid tumors
NCT03744468 [176]	358	Recruiting	Advanced solid tumor	TIM-3 LAG-3 PD-1	Drug: BGB-A425 Drug: Tislelizumab Drug: LBL-007	Evaluation of various combinations of BGB-A425 (Anti-TIM-3) and/or LBL-007 (Anti-LAG-3) with tislelizumab (Anti-PD-1) in patients with advanced solid tumors
NCT04810611 [177]	90	Recruiting	Myelodysplastic syndromes	TIM-3 TGF-β IL-1β	Drug: MBG453 Drug: NIS793 Drug: canakinumab	Phase I study of MBG453 (Anti-TIM-3), NIS793 (Anti-TGF-β), canakinumab (Anti-IL-1β) as single agents and in combinations of MBG453 + NIS793 and MBG453 + canakinumab in patients with lower risk MSD
NCT02817633 [178]	475	Recruiting	Neoplasms	TIM-3	Drug: TSR-022 Drug: Nivolumab Drug: TSR-042 Drug: TSR-033 Drug: Docetaxel Drug: Pemetrexed Drug: Cisplatin Drug: Carboplatin	Phase I study of TSR-022 (Anti-TIM-3) antibody in monotherapy and combined with other drugs in Patients With Advanced Solid Tumors (AMBER)
NCT03066648 [179]	242	Active, not recruiting	Leukemia Leukemia, myeloid Leukemia, myeloid, acute Myelodysplastic syndromes Preleukemia Bone marrow diseases Hematologic diseases Chronic myelomonocytic leukemia	TIM-3 PD-1	Drug: Decitabine Drug: PDR001 Drug: MBG453 Drug: Azacitidine	MBG453 (Anti-TIM-3) as a single agent or in combination with either Decitabine, Azacitidine, PDR001 (Anti-PD-1) or PDR001 and Decitabine and combination of Decitabine with PDR001
NCT02608268 [180]	252	Terminated	Advanced malignancies	TIM-3 PD-1	Drug: MBG453 Drug: PDR001 Drug: Decitabine	MBG453 (Anti-TIM-3) in monotherapy or in combination with in adult patients with PDR001 (Anti-PD-1) or Decitabine (chemotherapy) in patients with advanced solid tumors

### Anti-TIM-3 bispecifics

Majority of study trials regarding anti-TIM-3 bispecific focus on monotherapy. Bispecific antibodies share targets with monoclonal antibody combination therapies but differ in working mechanism. One antibody can bind two antigens and increase antitumor effectiveness [129]. LY3415244 is an anti-TIM-3/anti-PD-L1 bispecific antibody. Phase I study of the mentioned antibody revealed unexpected immunogenicity, which led to early study termination. Immunogenicity risk can be related to the presence of antidrug antibodies (ADA). Therefore, ADAs analysis should become part of the novel antibodies risk assessment [181]. Several phase I clinical trials of bispecific antibodies targeting TIM-3 are to be conducted. The examples of clinical trials involving anti-TIM-3 bispecific are shown in Table 2.

**Table 2:** Examples of clinical trials involving Anti-TIM-3 bispecific

Identifier	Patients number	Recruitment status	Condition or disease	Target	Therapy protocol	Short description
NCT03708328 [182]	134	Active, not recruiting	Solid tumors Metastatic melanoma Non-small cell lung cancer (NSCLC) Small cell lung cancer (SCLC) Esophageal squamous cell carcinoma (ESCC)	TIM-3/PD-1	Drug: RO7121661	Phase I study of single agent RO7121661 (Anti-PD-1/Anti-TIM-3) consists of 2 parts: dose escalation and expansion
NCT04931654 [183]	81	Recruiting	Carcinoma, non-small-cell lung	TIM-3/PD-1	Drug: AZD7789	Phase I/IIa study of AZD7789 (Anti-PD-1/Anti-TIM-3) antibody in participants with advanced solid tumors
NCT05357651 [184]	100	Recruiting	Solid tumor Lymphoma	TIM-3/PD-1	Drug: LB1410	Phase I study of a bispecific antibody LB1410 (Anti-TIM-3/Anti-PD-1)
NCT05216835 [185]	180	Recruiting	Relapsed or refractory classical Hodgkin lymphoma	TIM-3/PD-1	Drug: AZD7789	Phase I/II study of AZD7789 in patients with relapsed/refractory classical Hodgkin Lymphoma. Study consists of 2 parts: Dose Escalation and Expansion
NCT03752177 [186]	12	Terminated	Solid tumor	PD-L1/TIM-3	Drug: LY3415244	Study of LY3415244 (Anti-PD-L1/Anti-TIM-3) bispecific antibody in patients with advanced solid tumors

### Anti-TIM-3 cell-based therapies

Cell-based therapies are one of the latest treatments achieving tremendous therapeutic successes, making them the potential for treating many currently intractable diseases through extremely potent mechanisms of action [187]. Adoptive cell therapy offers significant possibilities for effective cancer immunotherapy. Chimeric antigen receptor (CAR) T cells are intensively researched to overcome the limitations in anti-tumor treatment, especially solid tumors.

In the study by He et al., bispecific for CD13 and TIM-3 CAR T cells (BissCAR T cells) were prepared using nanobodies obtained with the Sequentially Tumor-Selected Antibody and Antigen Retrieval (STAR) system [188]. The purpose of the developed cells was to eliminate acute myeloid leukemia (AML), which is characterized by the increased expression of CD13 and TIM-3. BissCAR T cells successfully killed CD13 + TIM-3 + AML stem cells with reduced toxicity in vitro and in vivo. The authors suspect that CD19 and TIM-3 BissCAR T are sufficient to eliminate AML, which also minimizes the sacrifice of the tissue with only CD13 expression. However, it was proven that TIM-3 CAR T failed to eradicate NB4-TIM-3 cells CD13 + TIM-3 + (AML stem cells model) in vivo. Research carried out by Jafarzadeh et al. proved that TIM-3 knockdown significantly increased the effectiveness of CAR T cell therapy [189]. The study investigated fully human second-generation anti-mesothelin CAR T cells, encoded short hairpin RNA (shRNA) sequences anti-TIM-3. Increased cytotoxicity and cytokine production (IFN-γ, TNF-α, and IL-2) during tested CAR T therapy were observed in ovarian and cervical cancer cell lines in vitro. The stable knockdown of TIM-3 reduced its expression, thus minimizing the negative impact on the progress in therapy. Therefore, the anti-tumor effectiveness of anti-mesothelin CAR T cells was considerably enhanced, promoting their usage in further research, especially in solid tumors.

The genetic ablation of TIM-3, PD-1, and LAG-3 in CD8 + T cells by the CRISPR/Cas9 system has been successfully used as an adoptive T cell therapy in derived murine melanoma cell line transfected with the ovalbumin protein (B16-OVA). The immune checkpoint receptors blockade has not affected cytokine expression in vitro or increased toxicity. Moreover, an extended lifetime of modified CD8 + T cells was observed compared to unmodified ones, which is related to improved anti-tumor activity [190].

Likewise, simultaneous knockdown of PD-1, Tim-3, and Lag-3 by shRNA cluster in Her2-specific CAR T cells (PTL-Her2-CAR-T) enhanced CAR T infiltration and anti-tumor effect. Furthermore, PTL-Her2-CAR-T presence intensified CD56 expression, consequently increasing its interaction. To investigate the effectiveness of downregulation, particular inhibitory receptors in various combinations (single or double) of Her2-CAR-T cells were tested. Based on the cytokines production and cytotoxicity, the most satisfying results were demonstrated for the synergistic effect of all three genes' knockdown [191].

## Conclusions

The immune checkpoint inhibitor TIM-3 is considered a highly promising target in novel anti-cancer therapeutic strategies that could soon provide a breakthrough in cancer treatment. Clinical studies indicate that anti-TIM-3 targeted therapies have gained great research interest in recent years as a tool to overcome certain limitations of conventional therapies potentially. As understanding of the functional properties of TIM-3 is still limited and requires further research, we reviewed the current state of knowledge on the structure, function, and expression of TIM-3 in various types of cancer, together with its correlation with overall prognosis. In light of a long-term perspective, identifying the role of TIM-3 in different types of tumors dependent on their microenvironment would create the possibility of applying a personalized therapeutic approach to cancer treatment.

## Data Availability

Not applicable.
